# Robot-Assisted Transaxillary Thyroid Surgery—Feasibility and Safety of a Novel Technique

**DOI:** 10.5041/RMMJ.10147

**Published:** 2014-04-28

**Authors:** Naomi Rabinovics, Raphael Feinmesser, Patrick Aidan, Yaniv Hamzany, Gideon Bachar

**Affiliations:** 1 Department of Otorhinolaryngology Head and Neck Surgery, Rabin Medical Center, Beilinson Campus, Petach Tikva, and Sackler Faculty of Medicine, Tel Aviv University, Tel Aviv, Israel; 2 Department of ENT Head and Neck surgery, The American Hospital, Paris, France

**Keywords:** Robot, thyroidectomy, transaxillary

## Abstract

Developments in technology have led to a rapid progress in robotic endocrine surgery applications. With the advent of minimally invasive techniques in thyroid surgery, robot-assisted transaxillary thyroid surgery (RATS) has emerged as one of the most promising approaches. Its main advantages are improved cosmetic outcome, avoiding cervical incisions, thereby increasing patient satisfaction, and improved visualization, arms articulations, and precision, resulting in fewer surgical complications. The main disadvantages are potential new injuries to the brachial plexus, esophagus, and trachea, longer operative time, and increased cost compared to conventional thyroidectomy. In skilled hands, RATS is a safe alternative to conservative thyroidectomy and should be presented to patients with aesthetic concerns. As with any new emerging technique, careful patient selection is crucial, and further evidence must be sought to confirm its indications over time.

## INTRODUCTION

Since the nineteenth century, when Kocher implemented the classical cervical thyroidectomy, little has changed in this procedure.^1^ When performed by experienced surgeons, the cervical approach is relatively short but unfortunately leaves a noticeable scar. Further advances in surgical instrumentation have introduced the minimally invasive thyroid surgery. The endoscopic thyroid surgery resulted in less morbidity and smaller surgical scars and developed into several different techniques.^2^ Nevertheless, the endoscopic cervical approach is surgically challenging since the neck is a very confined space and can be applied today to a small group of patients.

The non-cervical, remote access approaches originally developed primarily due to cosmetic considerations—poor wound healing of certain ethnic groups and the aversion in the Asian culture to neck scars.^3^ Ikeda et al. in 2000 were the first to develop the transaxillary endoscopic approach to the thyroid.^4^

With the introduction of the Da Vinci robot (Intuitive Surgical, Sunnyvale, CA, USA), some surgeons have recognized its potential advantages. The South Korean team from Seoul, led by Chung, pioneered the transaxillary approach to the thyroid gland in late 2007.^1^,^5^ The robotic-assisted transaxillary thyroid surgery (RATS) approach was first described in North America by Kupersmith and Holsinger in 2011,^3^ where body-build differs significantly from that of Koreans. Among the other robot-assisted thyroidectomy (RT) approaches (facelift approach, bilateral-axillary breast approach (BABA)), the transaxillary became the most popular. The initial RATS was performed via two incisions (axillary and anterior chest wall), but later the modification using a single axillary incision was described.^1^,^3^

The RATS has gained much popularity over the past 6 years, with several groups publishing their successful experience. However, since the conventional approach is safe, effective, and time-honored, some surgeons doubt the value of using robotic thyroid surgery and its clinical use.^6^

Although several eligibility criteria to RATS were described, no standard selection criteria have been established.^7^ Absolute contra-indications are previous neck surgery or irradiation, retrosternal thyroid extension, and advanced thyroid disease (invasion of trachea, esophagus, distant metastases). Relative contra-indications are patient co-morbidities, age, obesity, very large goiters, well-differentiated carcinomas with a diameter larger than 2 cm, lateral neck metastases, and previous ipsilateral shoulder dysfunction.^4^,^8^,^9^

## ADVANTAGES OF RATS

The most obvious advantage of RATS over conventional cervical thyroidectomy is that it eliminates the need for any cervical incision. This cosmetic aspect makes RATS appealing especially to young female patients and those with a tendency toward keloid formation.

The RATS has several technical advantages over the open and endoscopic approaches. First, the robotic system provides three-dimensional magnified visualization, which enables an easier identification of the recurrent laryngeal nerve (RLN) and parathyroid glands compared to the cervical approach. Second, it eliminates the natural surgeon tremor; and, third, it enables a wider range of motion through the robot’s endowrist and the articulations of the arms. All of these result in minimal complication rates and excellent cancer control and functional results. In addition, the improved visualization and surgical ergonomics provide for reduced musculoskeletal discomfort to the surgeon compared with open or endoscopic surgery.

RATS was found to yield better patient outcomes, including reduced pain and increased cosmetic satisfaction, as well as lower rates of paresthesia, postoperative voice change, and swallowing discomfort.^5^

## DISADVANTAGES OF RATS

On the other hand, due to the new approach to the surrounding anatomy and the loss of tactile sensation, RATS introduces potential new complications such as tracheal and esophageal injury. Very few studies accounted for such complications and then only in a minor way with no need to convert to open thyroidectomy.^1^ In addition, due to the ipsilateral arm position, there is a risk of brachial plexus neuropathy. This risk can be reduced by placing the arm in a flexed overhead 90 degrees position, thereby reducing the chance of stretching the nerves. Intra-operative monitoring of the ulnar, radial, and median nerves may further reduce the possibility of brachial plexus injury, by identification of any impending damage to these nerves and enabling the patient to be repositioned.^1^

Another disadvantage of RATS is the longer operative time due to the creation of the working space and the robot docking. However, several studies have examined the learning curves of the RT and have shown that increased experience led to decreased total operative time.^1^ RATS involves a steep learning curve, compared to the conventional approach. However, it has been demonstrated that compared to the endoscopic approach which requires 55–60 procedures, the RT required only 35–40 procedures.^5^ Another disadvantage of RATS is the limitation in the body habitus and BMI. While obese patients (BMI>30) make the operation (particularly the working space preparation) challenging, it has been demonstrated that, in skilled hands, this obstacle can safely be overcome.^1^,^10^,^11^

In terms of cost, the RT is a more expensive procedure compared to the open thyroidectomy, due to the cost of the equipment and the longer operative time. However, some studies have pointed out that RT eliminated the need for an additional surgical assistant, and, combined with the potentially shorter hospital stay and the expected decrease in the maintenance cost of the robot, this may eventually result in an equally cost-effective procedure.

## RATS IN PAPILLARY THYROID CARCINOMA

RATS is also performed in papillary thyroid carcinoma cases. In 2011 Lee et al. published their experience with RT on 1,043 patients with low-risk well-differentiated thyroid carcinoma. They showed that the RATS was feasible and offered outcomes similar to conventional and endoscopic thyroidectomies. This study included several surgeons, including junior ones, from a number of medical centers.^12^

The resection of the contralateral thyroid lobe in total or subtotal thyroidectomy is challenging via a single axillary incision. Therefore some surgeons doubted the surgical completeness of the RATS. Several studies investigated the completeness of the thyroidectomy, comparing it to conventional thyroidectomy using stimulated thyroglobulin levels, RAI uptake, and postoperative sonography. These studies ultimately demonstrated that the surgical completeness of RT is comparable to conventional thyroidectomy, if performed by experienced surgeons.^13^–^17^

## RATS EXPERIENCE

A meta-analysis comparing surgically related complications between robotic-assisted thyroidectomy (both BABA and RATS) and conventional open thyroidectomy summarized 11 studies, including 2,375 patients (1,536 of whom underwent RT), and concluded that robotic thyroidectomy had a longer operating time, longer hospital stay, and higher risk of temporary RLN injury than open thyroidectomy, but had comparable permanent complications and overall morbidity.^18^ Another meta-analysis published in 2012 by Jackson et al.^1^ summarized a total of nine studies with 2,881 patients, 1,122 of whom underwent RT. They conclude that RT is as effective as endoscopic and open thyroidectomy, with equivalent post-operative results, shorter hospitalization, and higher patient satisfaction. Lee et al. have also published their experience with 2,014 patients who underwent RATS, with a low complication rate of 1% for major complications (e.g. permanent RLN or brachial injury, conversion) and 19% for minor ones (transient hypocalcemia, seroma, etc.). Interestingly, this group also compared the surgeons’ perspectives on the musculoskeletal ergonomic parameters associated with RATS and endoscopic and open surgery. They concluded that RATS resulted in less neck and back discomfort than did the other approaches.^18^

RATS is being practiced mainly in South Korea and Europe and, to a smaller extent, in the US and Israel. Aidan et al. (personal communication; unpublished data) have performed, in Paris, France, over 190 RATS including 98 total thyroidectomies, 82 partial thyroidectomies, 10 parathyroidectomies, and 17 central node dissections. The total operative time for partial thyroidectomy was 142 minutes, and 170 minutes for a total thyroidectomy. They reported only 4 (2%) conversions to open surgery, 2 revision surgeries (1%), 1% permanent RLN injury, no permanent brachial plexus injury (4% were transient and resolved in 4–8 weeks), and no cases of permanent hypocalcemia (11% were transient). It should be noted that 55% of patients had large thyroid glands (whose volumes according to preoperative sonography or final pathology were over 20 mL).

The current Israeli experience with RATS in the Rabin Medical Center is very promising, with 20 cases of partial thyroidectomies (Table 1). RLN monitoring was implemented in all patients, and brachial plexus monitoring in the last five patients. In addition, patients were treated postoperatively with physiotherapy for the arm and shoulder. Hospital stay did not differ from conventional thyroidectomy patients, and neither did the amount of blood loss. There were no cases of esophageal or tracheal injuries. With careful patient selection and a detailed explanation of the possible complications, we found high rates of patient satisfaction.

**Table 1.** Characteristics of RATS Patients and Procedures at Rabin Medical Center.

**Variables**	**No.**
Sex: female/male	20 (100%)/0
Age, y (range)	42 (23–74)
Preoperative diameter of thyroid nodules, cm (range)	2.6 (0.5–4.2)
Pathology:	
Benign nodule	12 (60%)
Papillary carcinoma	7 (35%)
Follicular carcinoma	1 (5%)
Surgical time:	
working space	50 min
robot docking	15 min
console time	80 min
total procedure (from intubation to skin closure)	190 min
Complications:	
conversion to open	1 (5%)
permanent RLN injury	1 (5%)
permanent brachial plexus injuries	0

RLN, recurrent laryngeal nerve.

A newly reported use of the RATS for modified radical neck dissection (MRND) suggests that the precise movements and magnified 3D vision enable a meticulous and safe dissection with recovery of similar numbers of lymph nodes as an open procedure.^12^,^17^

## CONCLUSIONS

The cervical approach is currently the “gold standard” procedure for thyroidectomy. However, in skilled hands, RATS is considered a safe alternative and should be presented to patients, especially those with aesthetic concerns. Terris stated that “We are in a period where one size no longer fits all”^3^—there is a diversity of different approaches, and the surgeon should tailor the procedure to the patient’s disease, general state, and desires. It is the surgeon’s obligation to introduce the patient to the different surgical options and consult him on the most appropriate one. With increasing experience and continued improvement in the robotic technology, the indications for RT will continue to evolve.^6^ The use of the robot for neck dissection via a transaxillary incision will continue to evolve and the indications to perform RATS will continue to expand. RATS should probably be performed in high-volume centers, by skilled surgeons. As with any new emerging technique, careful patient selection is crucial, and further evidence must be sought to confirm its indications over time.

## Abbreviations:

RATS: robot-assisted transaxillary thyroid surgery
RLN: recurrent laryngeal nerve
RT: robot-assisted thyroidectomy.
